# Deep Residual Network in Network

**DOI:** 10.1155/2021/6659083

**Published:** 2021-02-23

**Authors:** Hmidi Alaeddine, Malek Jihene

**Affiliations:** ^1^Faculty of Sciences of Monastir, Electronics and Microelectronics Laboratory, Monastir University, Monastir 5000, Tunisia; ^2^Higher Institute of Applied Sciences and Technology of Sousse, Sousse University, Sousse 4000, Tunisia

## Abstract

Deep network in network (DNIN) model is an efficient instance and an important extension of the convolutional neural network (CNN) consisting of alternating convolutional layers and pooling layers. In this model, a multilayer perceptron (MLP), a nonlinear function, is exploited to replace the linear filter for convolution. Increasing the depth of DNIN can also help improve classification accuracy while its formation becomes more difficult, learning time gets slower, and accuracy becomes saturated and then degrades. This paper presents a new deep residual network in network (DrNIN) model that represents a deeper model of DNIN. This model represents an interesting architecture for on-chip implementations on FPGAs. In fact, it can be applied to a variety of image recognition applications. This model has a homogeneous and multilength architecture with the hyperparameter “L” (“L” defines the model length). In this paper, we will apply the residual learning framework to DNIN and we will explicitly reformulate convolutional layers as residual learning functions to solve the vanishing gradient problem and facilitate and speed up the learning process. We will provide a comprehensive study showing that DrNIN models can gain accuracy from a significantly increased depth. On the CIFAR-10 dataset, we evaluate the proposed models with a depth of up to *L* = 5 DrMLPconv layers, 1.66x deeper than DNIN. The experimental results demonstrate the efficiency of the proposed method and its role in providing the model with a greater capacity to represent features and thus leading to better recognition performance.

## 1. Introduction

With the increase in the depth of the DNIN model, a problem of degrading the training precision has been unexpectedly exposed; the accuracy is saturated and then degrades rapidly. This degradation is not caused by overadjustment. It seemed clear that adding more Deep MLPconv (DMLPconv) layers to the DNIN models results in a higher training error, as reported in [1]. Generally, it has been shown that every fraction of the improved accuracy is costly in terms of the number of layers; hence, the formation of very deep networks poses problems such as reduced reuse of features during forward propagation, exploding/vanishing gradients making these networks very slow to form. However, several techniques are exploited to solve this problem. We note among them batch normalization [2], stochastic depth [3], well-designed initialization strategies [4, 5], better optimizers [6], skipping connections [7, 8], knowledge transfer [9, 10], layered training [11], normalized initialization [5, 12, 13], and residual blocks [14]. Experiments show that residual blocks [14, 15] were comparatively good or/and better than these various techniques and indicate that a deeper model should not produce higher training error than its shallower counterpart. The depth of the last residual deep networks [14] is evolved up to thousands of layers while improving their performance. They have had great success and reached the state of the art in several benchmarks. In this article, we address the degradation problem by introducing an efficient deep neural network architecture for computer vision, deep residual network in network, which takes its name from the deep network in the network article [1] in conjunction with the famous “deep residual learning for image recognition” [14]. The advantages of the architecture are experimentally verified on the CIFAR-10 classification challenges. The contributions of this work are as follows:

- We propose a new residual architecture for the DMLPconv layers which allows to have DrNIN models with considerably improved performance
- We propose a new way to use batch normalization and dropout in the DrNIN model in order to regularize and normalize them properly and avoid overfitting during training
- We present a detailed experimental study of multilength deep model architectures that examines in depth several important aspects of DrMLPconv layers
- Finally, we show that our proposed DrNIN architectures obtain interesting results on CIFAR-10 considerably improving the precision and training speed of DrNIN

The rest of this article is organized as follows: Section 2 presents an overview of related work.  Section 3 bears the strategy. Experimental results are presented and discussed in Section 4. The advantages and limitations of DrNIN are presented in Section 5. The work is concluded in Section 6.

## 2. Related Works

**Figure 1:**
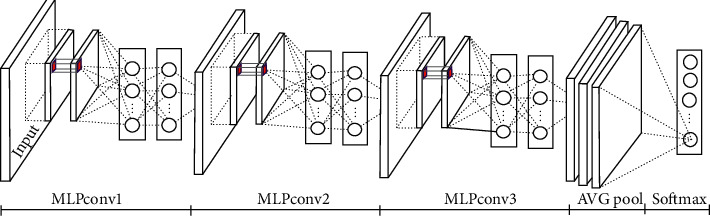
Deep network in network.

Generally, various techniques are used to improve the performance of CNNs in terms of precision or parameters and computational complexity such as increasing the depth [14, 16–20], changing the filter type [1, 21, 22], increasing the width [19, 23], number of units of each layer and/or the number of feature maps (channels) [23, 24], modification of convolution parameters [25–29] or pooling [30–38], changing the activation function [1, 39, 40], and reducing the number of parameters and resources [1, 27, 41]. In CNN, the computation in the convolutional layer is based on the simple linear filter. However, changing the filter type is an important step to develop efficient CNNs. Using a nonlinear and more complex filter, such as an MLP filter, can generate more interesting results than using a simple linear filter [1, 21]. Several architectures were based on this principle such as [1, 21, 42]. NIN [21] adopts a nonlinear filter: the multilayer perceptron (MLP) with a rectified linear unit (ReLU) used as an activation function. In [1], DNIN model directly modifies NIN [21] in the sense of convolutional layer. It is represented in a three-layer stacking DMLPconv, which consists of two convolutional layers of size 3 × 3 and an eLU unit, used as an activation function instead of ReLU. By incorporating micronetwork, DNIN [1] also increases depth. The depth of DNIN [1] is the same as that of NIN [21] and shares the same number of convolutional kernels. DNIN [1] is illustrated in Figure 1. Our proposed model is closely related to DNIN [1] and is based on increasing depth. One of the main differences between CNNs and classical neural networks is the depth. AlexNet [27] contains eight learned layers (five convolutional layers and three fully connected ones) without taking into account the pooling layers. AlexNet [27] is the first architecture to use the rectified linear unit (ReLU) for the activation function in order to improve the rate of convergence by reducing the vanishing gradients problem. In VggNet [17], the depth ranges from 11 up to 19; VGG with 16 layers has a homogeneous and regular structure. GoogLeNet [24] introduced by Christian Szegedy et al. is a CNN with a depth of 22 layers. In ResNet [14], a residual block is proposed to facilitate the formation of very deep networks. The principle of these blocks rests upon including a link around each two convolution layers by adding the diverted original data and their results from the convolution function. This architecture is similar to GoogLeNet [24] in terms of the use of a global average pooling followed by the classification layer. In [39], the Maxout network delivers a solution to the vanishing gradients problem. Maxout units have been designed to facilitate and enhance dropout layers. They were originally intended to replace the ReLU functions. In [40], a Maxout network in Maxout network (MIM) model incorporating a maximum number of units that are stacked in a MIM block is proposed. The model [40] is more complex than the Maxout network [39]. In [43], quadratic units were given in order to improve the robustness. Furthermore, the authors in [44] succeeded in forming quadratic units. In [45], the authors proposed logarithmic activation functions. From these literatures, we considered already accomplished approaches and already carried out experiments in order to improve the original architecture of DNIN [1] in order to obtain a better precision where we can apply the residual learning framework to the different layers MLPconv, and reformulate convolutional layers as residual learning functions.

## 3. Proposed Model

### 3.1. Deep Residual MLPconv

**Figure 2:**
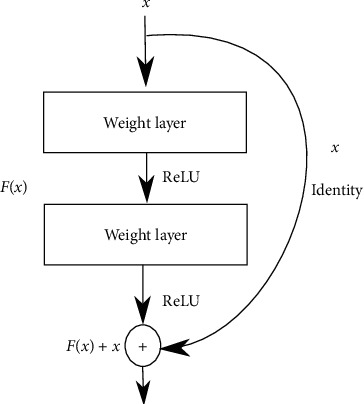
Residual learning: a building block.

Compared to the original DNIN architecture [1], a residual function block is applied inside the Deep MLPconv layers. The new layer is named Deep residual MLPconv (DrMLPconv). The residual block (Figure 2) with identity mapping is described in subsection 3.2 of [14] and its formula is represented as follows:

(1) $$\begin{array}{c} x_{l+1}=x_{l}+F\left(x_{l},W_{l}\right), \end{array}$$

where *x*_*l*+1_ and *x*_*l*_ are the input and output of the *l*^th^ unit in the network, F is a residual function, and *w*_*l*_ are parameters of the block. The residual network consists of the residual blocks stacked sequentially.

Small filters of 3 × 3 size have been shown to be very effective in several works including [14, 17, 23]; they are almost exploited in works published after VggNet [17]. In our work, we do not plan to use filters larger than 3 × 3, compared to the original “Deep MLPconv” architecture [1]. Moreover, for all DrMLPconv layers, the numbers of convolutional kernels MLP-2 are the same. MLP-1 is equivalent to 96.  Table 1 describes the number of kernels for DrMLPconv.

**Figure 3:**
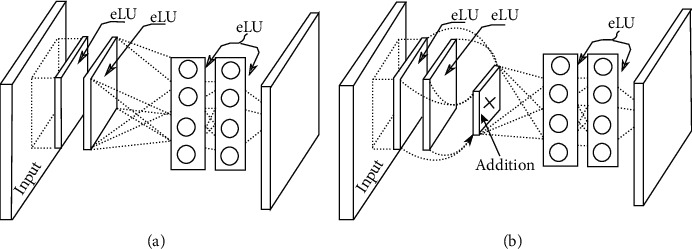
(a) A schematic example of DMLPconv layer, (b) a schematic example of “basic” DrMLPconv.

The new base structure of the DrMLPconv is based on a residual block [14], a multilayer perception (with a depth of two layers) which is described as a complex nonlinear filter. Note that basic DMLPconv, as shown in Figure 3(a), consists of two convolution layers of size 3 × 3, MLP layers. These different layers are followed by an eLU activation.  Figure 3 shows, respectively, a schematic example of basic DrMLPconv and DMLPconv [1].

Let DrMLPconv (*X*) be the DrMLPconv layer, where *X* is a list of the layers used in the structure. For example, DrMLPconv (3, R) denotes the basic DrMLPconv layers with a residual block applied to two convolution layers of size 3 × 3. All the configurations of the DrMLPconv layer are equipped with the eLU nonlinearity [41]; DrMLPconv (3, *R*, BD) denotes the structure DrMLPconv (3, R) with the normalization and regularization layers (BD). The different structure of our DrMLPconv is shown in Table 2.

### 3.2. The Structure of DrNIN

We describe our various configurations of DrNIN models for CIFAR-10. In these model configurations, the convolutional layers mostly have 3 × 3 filters and follow two simple design rules: first, the layers that participate in the residual block have the same size of output function feature map and the same number of filters; second, the exploitation of a pooling layer which is generally inserted periodically between a stack of *L* successive DrMLPconv layers of an architecture in order to preserve the temporal complexity by layer. In architectural design, pooling layers are another important concept that allows great gains in computing power due to the reduction in the spatial size of an image. We do a subsampling using the max pooling layers of size 3 × 3 which have a stride of 2 (3 × 3/ST.2). The network ends with a global average pooling layer and a softmax layer. The global average pooling layer filter size depends on the hyperparameter “L.” Table 3 summarizes the sizes of these global average pooling layers.

**Figure 4:**
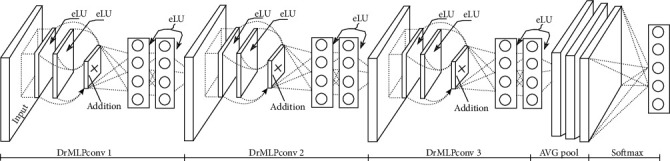
A DrNIN with 3 DrMLPconv (3, R).

Our configurations are captured in an RGB image of fixed size equal to 32 × 32. The image is passed through a layer stack that is built with variable and complex structures.  Figure 4 illustrates an example of the DrNIN model composed of three DrMLPconv (3, R) layers.

The overall structure of DrNIN generally consists of the *L* layer DrMLPconv.  Table 4 shows the overall structure of DrNIN for three different hyperparameters. In addition, it shows the output sizes after each layer used in the model.

#### 3.2.1. Dropout and Batch Normalization in DrNIN

The use of regularization represents a solution to avoid overlearning. A batch normalization [2] is already applied for DrNIN in order to provide a regularization effect. This layer is localized after the convolutional layers, and before the nonlinearity. Using this layer makes DrNIN more resistant to bad initialization. Moreover, it eliminates the need for the use of dropout layer [46]. Dropout layer [46] is an extremely efficient regularization technique that complements the L1, L2 regularization methods which are used to monitor the ability of neural networks to prevent overlearning. They are widely exploited for the purpose of introducing regularization into deep neural networks and to prevent neural networks from overadjusting. The purpose of this technique is to randomly remove units or connections in order to prevent the units from adapting to them, which can improve the classification accuracy in many studies [1, 21, 42]. This technique proves that during training these layers ultimately improve generalization by randomly skipping a selectable percentage of their connections. When training, there are neurons that do not contribute to the propagation and do not participate in the backpropagation. At the time of the test, all neurons are used but their outputs are multiplied by the probability. Generally, the probability of 0.5 is the most used. The downside of this layer is that it roughly doubles the number of iterations needed to converge. Using this layer with a probability of 0.5 reduces the error rate to almost 2% for almost all configurations. Note that the dropout layers [46] are added between the DrMLPconv layers and after the pooling layers.

#### 3.2.2. The Effect of Data Augmentation in DrNIN

Data augmentation [47] is defined as an augmentation process that significantly improves the quality of predictions by artificially increasing the data volume for training the model without the need to collect new data, that is, creating new data from existing data. Data augmentation techniques can consist of rotations, distortions, cropping, color changes, adding noise, padding, and horizontal flipping commonly used to train large neural networks. Exploitation of this confirming layer shows a positive effect in reducing the classification test error and automatically leads to significantly better results than learning without exploiting this layer. In addition, experimental results show that the DrNIN model with batch normalization [2] achieves higher precision than a DrNIN without this normalization layer.

## 4. Experimental Results

### 4.1. Overview

For the training of our model, we have adapted the same training details exploited by [1] to form our configurations. In addition, we have adapted the same procedure for initializing neural weights and biases in all convolutional layers as well as MLP layers. For the learning rate, it was initialized to 0.01 and divided by 10 three times before the end of the training at times 35, 55, and 90. We trained the network for about 195 cycles on the CIFAR-10 dataset in an Intel Xeon Processor E5-2620 v4, 64 GB DDR4-2400, 8 cores, 16 threads. The design and implementation of this model is done using the TensorFlow deep learning library to classify and recognize images. The CIFAR-10 (Canadian Institute for Advanced Research) dataset consists of 60,000 images grouped into 10 image classes with 6,000 images in each class. This collection of images is commonly used to train machine learning and computer vision algorithms. In this database, all the images are RGB images of size 32 × 32. The dataset is divided into five training packages and one test package, each containing 10,000 images. The test lot contains exactly 1000 images selected at random from each class. The training packages contain exactly 5000 images of each class. The classes are completely exclusive of each other. There will be no overlap between automobiles and trucks. Unlike the MNIST dataset, the objects in these classes are much more complex in nature and extremely varied. If we look at the CIFAR-10 dataset, we realize that there is not just one type of bird or cat. The class of birds and cats contains many types of birds and cats that vary in size, color, magnification, different angles, and different poses. In the following, we evaluate our different configurations proposed on this benchmark dataset.

### 4.2. The Performances of Different Configurations

The experimental results shown in Table 5, on the CIFAR-10 datasets, show the test accuracy rates for all of the proposed DrNIN configurations. These experimental results which were obtained by calculating the average over 5 runs with mini lot size equivalent to 128 also demonstrate the effectiveness of the proposed idea of reformulating convolutional layers as residual learning functions.

Moreover, they show that the DrNIN offers better results than the different DNIN configurations [1], which are, respectively, 88.25%, 90.63%, and 92.54%.  Table 6 shows the difference between the test accuracy of different similar configurations of DNIN [1] and DrNIN (*L* = 3). The test accuracy of the basic DrNIN configuration exceeds the basic DNIN configuration with 0.18%, and the DrNIN configuration with the normalization and regularization layers exceeds the DNIN configuration with the normalization and regularization layers with 0.57%. Finally, DrNIN with data augmentation exceeds DNIN [1] with the same layer with 0.32%. It is recalled that the DNIN [1] delivers a precision equivalent to 90.44% by using 4 DMLPconv layers with dropout layers [46] without using batch normalization layers [2].

**Figure 5:**
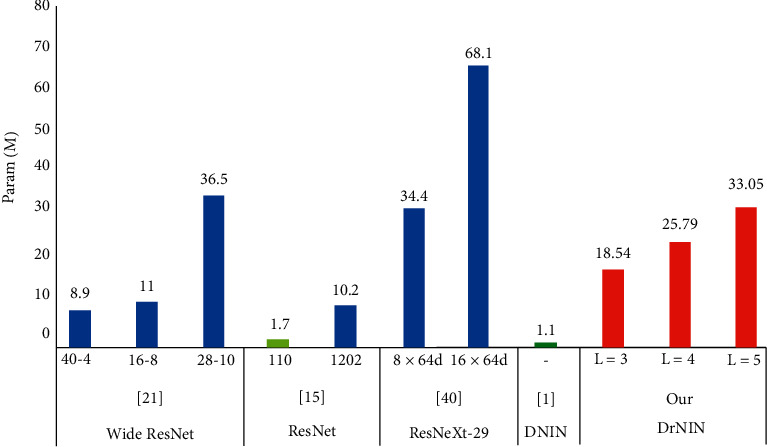
The parameters of the architectures already completed. The parameters (*M*) of our models are in red.

In terms of parameters, our model consumes 18.54 M for a configuration with a hyperparameter equivalent to 3 (*L* = 3). For a configuration of 4 DrMLPconv (*L* = 4), the model uses 25.79 M and 33.04 M for a configuration with *L* = 5. It offers a number of parameters superior to the WRN (16–8; 40–4) and ResNet (110, 1202) models despite their depth and width. For example, the DrNIN model with a hyperparameter equivalent to 3 (*L* = 3) consumes 16.85 times more parameters than DNIN [1], 10.90 times more than ResNet-101, and 0.27 times more than ResNeXt-29 (16 × 64d).  Figure 5 shows the number of parameters consumed from architectures already completed.

DrNIN provides classification precision that allows it to have a well-localized location between multiple baselines. Moreover, the experimental results show that the exploitation of the data augmentation layer [47] or/and the batch normalization layer [2] produces a useful effect in reducing the error of the classification test.  Table 7 represents a comparison between the proposed model and the state of the art on the CIFAR-10 database with/without the use of data augmentation. The results of our work are presented with mini lot size equivalent to 128 and by calculating the average of 5 runs.

### 4.3. Visualization of Weights

**Figure 6:**
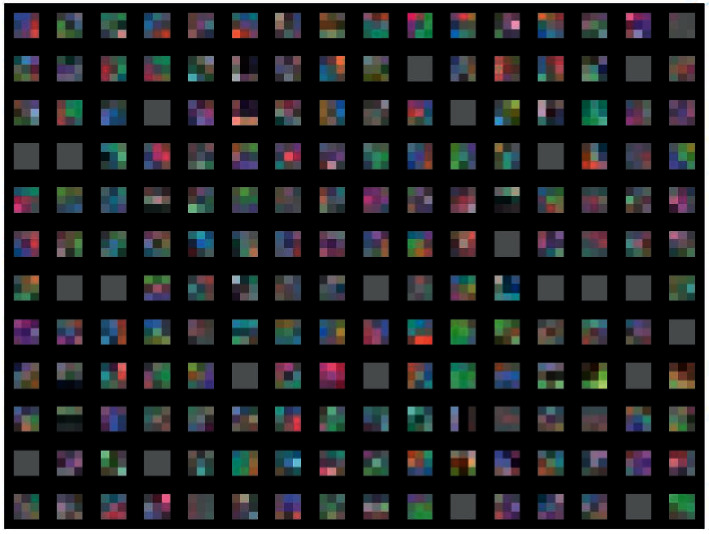
192 convolution cores of size 3 × 3 learned by the first convolution layer of the DrMLP (3, R, BD, D).

The convolution layer always constitutes at least the first layer and its goal is to identify the presence of a set of features in the images received as input. Viewing the weights of the first convolutional layer is most preferable since it looks directly at the raw pixel data. In the following, we visualize the weights of 192 convolutional kernels of size 3 × 3 learned by the first convolution layer on the 32 × 32 input images for the first convolution layer of DrMLPconv (3, *R*, BD) in Figure 6.

## 5. Advantage and Limitations

The proposed DrNIN model provides an interesting and competitive test precision which exceeds the precision of other models based on nonlinear filters such as [1, 21, 39, 42] and which allows it to occupy an important place between the various works reported in the literature. DrNIN provides interesting test errors against the baseline. The importance of DrNIN also stems from its homogeneous structure which makes it very suitable for implementation as a hardware accelerator in FPGAs or integration as an image recognition system in embedded systems applications. However, DrNIN incorporates drawbacks and limitations which mainly reside in the number of DrMLPconv layers “L” and the number of convolution kernels. This negatively affects the number of parameters, computational complexity, and memory.

## 6. Conclusion

In this paper, a new deep residual network in network (DrNIN) model for image classification is proposed. In this model, a new nonlinear DrMLPconv filter is used. This layer is based on a residual block applied to very small convolutional filter sizes (3 × 3) to accelerate learning model. The use of these layers leads to an improvement in the classification precision. In addition, a proposed, detailed study and experimental DrNIN model is presented describing with details the effect of different layers on improving accuracy. The results are described as acceptable compared to other architectures tested on the CIFAR-10 datasets and once again confirm the importance of residual block on increasing depth and improving classification accuracy. Future work should focus on designing new versions of CNN models that can achieve or exceed level accuracy of this proposed model requiring shorter training time with less parameter consumption.

## Figures and Tables

**Table 1:** The numbers of kernels for all DrMLPconv.

Layers	Conv 3 × 3	MLP-1	MLP-2
Number	192	160	192

**Table 2:** The configurations of DrMLPconv.

Layer	DrMLPconv (X)	
(*X*)	DrMLPconv (3, R)	DrMLPconv (3, E, BD)
Conv-1	3 × 3 × 192/st. 1/pad 1/eLU	3 × 3 × 192/st. 1/pad 1/eLU/BN
Conv-2	3 × 3 × 192/st. 1/pad 1/eLU	3 × 3 × 192/st. 1/pad 1/eLU/BN
MLP-1	1 × 1 × 160/st. 1/pad 0/eLU	1 × 1 × 160/st. 1/pad 0/eLU/BN
MLP-2	1 × 1 × 192/st. 1/pad 0/eLU	1 × 1 × 192/st. 1/pad 0/eLU/BN

**Table 3:** The filter size of the global average pooling layers.

Hyperparameters	3	4	5
Global average pooling size	8 × 8	4 × 4	2 × 2

**Table 4:** The structure of DrNIN.

Layer name	Output size		
-	*L* = 3	*L* = 4	*L* = 5
DrMLPconv-1	32 × 32	32 × 32	32 × 32
Max-pool	16 × 16	16 × 16	16 × 16
DrMLPconv-2	16 × 16	16 × 16	16 × 16
Max-pool	8 × 8	8 × 8	8 × 8
DrMLPconv-3	8 × 8	8 × 8	8 × 8
Max-pool	—	4 × 4	4 × 4
DrMLPconv-4	—	4 × 4	4 × 4
Max-pool	—	—	2 × 2
DrMLPconv-5	—	—	2 × 2
Global average pooling	1 × 1	—	—

**Table 5:** Test error (%) of DrNIN on CIFAR-10.

X	#Depth	CIFAR-10
(3, R)	3	11.57%
	4	11.25%
	5	11.11%

(3, *R*, BD)	3	09,88%
	4	09,37%
	5	09,03%

(3, *R*, BD, D)	3	07,34%
	4	07,28%
	5	07,21%

**Table 6:** CIFAR-10 test error. A comparison between DNIN [1] and DrNIN for *L* = 3.

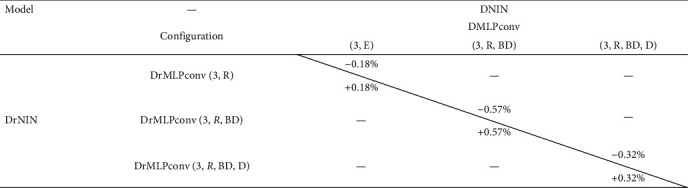

**Table 7:** CIFAR-10 test error.

Ref	Method	Error (%)
*Without data augmentation*		
[30]	Stochastic pooling	15.13
[39]	Maxout network (*k* = 2)	11.68
[2]	NIN	10.41
[1]	DNIN	9.37
Our	DrNIN (*l* = 5)	9,03
[40]	MIM (*k* = 2)	8.52 ± 0.20

*With data augmentation*		
[39]	Maxout network (*k* = 2)	9.38
[2]	NIN	8.81
[1]	DNIN	7.46
Our	DrNIN (*l* = 5)	7.21
[14]	ResNet	6.43
[23]	Wide ResNet (28, 10)	3.89
[48]	ResNeXt	3.58

## Data Availability

The data used to support the findings of this study are included within the article.

## Abbreviations

CNN: Convolutional neural network
NIN: Network in network
DNIN: Deep network in network
DrNIN: Deep residual network in network
MLP: Multilayer perceptron
DMLPconv: Deep MLPconv
DrMLPconv: Deep residual MLPconv
ReLU: Rectified linear unit
eLU: Exponential linear unit.
